# Effect of diet-induced weight loss on iron status and its markers among young women with overweight/obesity and iron deficiency anemia: a randomized controlled trial

**DOI:** 10.3389/fnut.2023.1155947

**Published:** 2023-05-22

**Authors:** Naseem Mohammad Alshwaiyat, Aryati Ahmad, Hamid Ali Nagi Al-Jamal

**Affiliations:** ^1^School of Nutrition and Dietetics, Faculty of Health Sciences, Gong Badak Campus, Universiti Sultan Zainal Abidin, Kuala Nerus, Terengganu, Malaysia; ^2^School of Biomedicine, Faculty of Health Sciences, Gong Badak Campus, Universiti Sultan Zainal Abidin, Kuala Nerus, Terengganu, Malaysia

**Keywords:** weight loss, obesity, iron status, chronic inflammation, hepcidin, iron deficiency anemia

## Abstract

**Introduction:**

Obesity and iron deficiency are prevalent health problems that affect billions of people all over the world. Obesity is postulated to relate to iron deficiency via reduced intestinal iron absorption due to increased serum hepcidin level, which is mediated by chronic inflammation. Weight loss in individuals with overweight or obesity and iron deficiency anemia is believed to be associated with an improvement in iron status however the evidence from clinical trials is scarce. This study was conducted to evaluate the effect of diet-induced weight loss on iron status and its markers among young women with overweight/obesity and iron deficiency anemia.

**Methods:**

The study design was a single-blinded, randomized controlled trial with two parallel arms (weight loss intervention vs control). Study participants were recruited using the convenience sampling method through public advertisements posted and disseminated through social media. Interested and potential participants were asked to visit the Diet Clinic for eligibility screening. A total of 62 women were recruited and randomized into weight loss intervention and control group. The intervention duration was three months. The intervention group received individual consultation sessions with the dietitian and tailored energy-restricted diets. Physical activity levels, dietary intake, anthropometric measurements and clinical markers were measured at baseline and end of the trial.

**Results:**

There was a significant decrease (*p* < 0.001) in body weight of the intervention group (-7.4 ± 2.7 kg) that was associated with significant improvements in iron status and its markers (*p* < 0.01). The intervention group experienced a significant increase in hemoglobin (0.5 ± 0.6 g/dL), serum ferritin (5.6 ± 5.8 ng/mL), and serum iron (13.0 ± 16.2 µg/dL), and a significant decrease in high-sensitivity C-reactive protein (-5.2 ± 5.6 mg/L), and serum hepcidin level (-1.9 ± 2.2 ng/mL) at the end of the trial.

**Conclusion:**

Our findings indicate that diet-induced weight loss among participants was associated with an improvement in iron status and its related clinical markers.

**Clinical Trial Registration:**

[https://www.thaiclinicaltrials.org/show/TCTR20221009001], identifier [TCTR20221009001].

## 1. Introduction

Obesity and iron deficiency are serious public health issues affecting billions of people worldwide (1, 2). Obesity is widely prevalent among adults and associated with high morbidity and mortality rates due to adverse health effects caused by excessive fat accumulation in the body, including elevated serum lipids (3–5). Concurrently, iron deficiency is still one of the most prevalent nutritional deficiency problems at the global level (2). This deficiency will lead to iron deficiency anemia (IDA), a critical health problem, which adversely affects cognitive function, physical performance, and work productivity (6). Previously, iron deficiency has been linked to pediatric and adulthood obesity, in which obesity is considered an emerging risk factor for iron deficiency incidence (7, 8). The connection between obesity and iron deficiency could be explained by the state of low-grade chronic inflammation in obesity, which stimulates the expression of hepcidin, a key regulator of iron homeostasis (9, 10).

Young women are susceptible to weight gain and become overweight or obese due to many factors, such as unhealthy dietary patterns, sedentary lifestyles, and pregnancy (11). At the same time, young women are also at high risk of iron deficiency as their dietary iron requirements are higher than older women due to menstrual losses (12). Dieting is common among women with overweight or obesity, particularly at this stage of life (13). Energy-restricted diets have long-term beneficial health effects, but can negatively affect dietary iron intake (14–16).

Several studies have reported that individuals with overweight and obesity were associated with lower iron status and elevated systematic inflammation and serum hepcidin compared to those with normal body weight (17–26). However, very limited studies investigated the effect of weight loss on iron status, systematic inflammation, and serum hepcidin among individuals with overweight or obesity, especially in clinical trials (27). Therefore, this study was conducted to evaluate the effect of diet-induced weight loss on iron status, chronic inflammation, and serum hepcidin level among overweight or obese young women with IDA.

## 2. Methods

### 2.1. Study design and participants

This study was a single-blinded, randomized controlled trial (RCT) with two parallel arms design (weight loss intervention vs. control). The study was conducted at a private diet clinic (Gharaibeh Diet Clinic, Ajloun, Jordan). The study protocol was approved by Human Research Ethics Committee at Universiti Sultan Zainal Abidin, Terengganu, Malaysia (UniSZA/UHREC/2020/172) and Ajloun Health Directorate, Ministry of Health, Ajloun, Jordan (No.: 22/8/140). The inclusion criteria were young adult Jordanian women (18–30 years)-with Arab ethnicity, being overweight [body mass index (BMI) = 25–29.9] or obese (BMI ≥ 30), and diagnosed with mild or moderate IDA [hemoglobin = 8.0–11.9 g/dL, mean corpuscular volume (MCV) < 80 fL and serum ferritin ≤30 ng/mL]. The exclusion criteria included symptomatic patients, pregnant or lactating within the past 12 months, presence of any chronic disease or significant medical condition, being vegetarian, tobacco smoker, alcohol drinker, or drug abuser, had unstable body weight within the past 6 months (weight change ≥3% of initial body weight), irregular menstrual cycle within the past 12 months, undergone bariatric surgery, or full or partial hysterectomy, donated blood or history of hemorrhage within the past 6 months, consumption of iron supplement within the past 6 months, or any other dietary supplement within the past 3 months, and use of medications that may influence weight, iron, or inflammatory status within the past 3 months such as contraceptive medication. Study participants were recruited using the convenience sampling method through public advertisements posted and disseminated through social media. Interested and potential participants were asked to visit the Diet Clinic for eligibility screening. Informed consent was obtained from all participants who met the inclusion criteria following the Helsinki Declaration prior to randomization. Block randomization was handled by an independent collaborator with an equal allocation using a computer-generated randomization schedule. The allocation was concealed until the intervention started. The allocation sequence was concealed from the researcher and participants in sequentially numbered, opaque, sealed, and stapled envelopes. The study randomization was blinded for measurers and data collectors. All methods were performed in accordance with the relevant guidelines and regulations. In this trial, exposure was diet-induced weight loss, while primary outcomes were changes in iron status markers, high-sensitivity C-reactive protein (hsCRP), and serum hepcidin. No important changes to methods or trial outcomes after trial commencement were applied.

### 2.2. Weight loss intervention

The intervention duration was 3 months. The intervention group received individual consultation sessions with the dietitian on days 0, 15, 30, 45, 60, and 75 for 30 min. During the consultations, participants received tailored energy-restricted diets, i.e., energy requirement with a deficit of 500 kcal with 50, 30, and 20% of daily energy from carbohydrate, fat, and protein, respectively, education about iron-rich dietary sources, and method of recording food intake. The weight loss target was 1–2 kg for 2 weeks. The serving size of foods included in the diet was based on American food lists (28), and Jordanian food lists (29, 30). The control group was asked to continue on habitual dietary patterns throughout the participation period.

### 2.3. Data collection

Data were collected at baseline (day 0) and the end of the trial (day 90). Sociodemographic and physical activity data were collected using a questionnaire during face-to-face interviews. Physical activity levels were assessed using the Global Physical Activity Questionnaire (GPAQ), which has acceptable reliability and validity for measuring adult physical activity levels (31, 32). Physical activity was categorized into three levels: low, moderate, and high (33, 34). Dietary intake was measured using 7-day food record method. Participants were required to record all foods and beverages consumed during the day in a specific form for 7 consecutive days preceding the diet clinic visit at baseline and during the study preceding each follow-up visit. Nutrition analysis software (Food processor, version 11.9, ESHA Research, Salem, OR, United States) was used to determine average daily nutrient intake.

### 2.4. Anthropometric measurements

Participants’ height, weight, waist circumference, hip circumference, and body fat percentage were measured by the dietitian in the morning after overnight fasting using standard procedures. Height was measured to the nearest 0.1 cm using a calibrated stadiometer (Seca 213, Germany) in the standing position without shoes. Weight was measured to the nearest 0.1 kg using a calibrated digital weight scale (Seca 876, Germany) while wearing minimal clothes without shoes. The BMI was determined by dividing the weight (kg) by squared height (m^2^) and classified into overweight (25–29.9 kg/m^2^) or obese (≥30 kg/m^2^) (35). The waist and hip circumferences were measured to the nearest 0.1 cm using anthropometric tape (Seca 203, Germany). The waist-hip ratio (WHR) was calculated by dividing the waist circumference by the hip circumference. The body fat percentage of participants was measured using a bioelectrical impedance analysis device (Tanita body composition monitor, BC-601F, Tokyo, Japan) according to the manufacturer’s instructions.

### 2.5. Blood analysis

Hematological and biochemical assays were conducted at a private certified medical laboratory (Ajloun Medical Labs, Ajloun, Jordan). Blood samples were collected from participants in the morning after overnight fasting on days 0 and 90. Complete blood count (CBC), including red cell (RBC) count, hemoglobin, hematocrit, MCV, mean corpuscular hemoglobin (MCH) and mean corpuscular hemoglobin concentration (MCHC), serum iron, total iron binding capacity (TIBC), serum ferritin, and hsCRP were measured according to standard medical laboratory procedures. Transferrin saturation (TS) was calculated by dividing serum iron on TIBC multiplied by 100%. Serum hsCRP was evaluated by sandwich immunodetection method using an AFIAS-6 automated immunoassay analyzer (Boditech Med Inc., Chuncheon-si, South Korea). Serum hepcidin level was determined using a human hepcidin immunoassay ELISA kit (Quantikine ELISA DHP250, R&D Systems, Minneapolis, MN, United States) according to the manufacturer’s instructions.

### 2.6. Statistical analysis

The sample size was calculated using G^*^Power software, Version 3.1.9.4, assuming that the test family is *t*-tests, the statistical test is the difference between two independent means (two groups), the tails are two, the effect size is equal to 0.80, the level of significance is equal to 0.05, and the power is equal to 0.80 (36). The total calculated sample size was 52 participants (26 participants in each group). After adding 20% to account for possible attrition, the required sample size was 62 participants (31 participants in each group).

IBM SPSS Statistics for Windows (Version 26, United States) was used for data analysis. Changes in dietary intake data were calculated by subtracting the during-intervention value from the baseline value (change value = during-intervention value−baseline value). Changes in anthropometric measures, iron status markers, hsCRP, and serum hepcidin were calculated by subtracting the post-intervention value from the baseline value (change value = post-intervention value−baseline value). The normality of data was assessed by visual inspection of histograms and the Shapiro–Wilk test. Categorical variables were presented as numbers and percentages. Continuous variables were presented as means and standard deviations. Chi-squared test was conducted to determine differences in categorical variables between the intervention group and the control group. Wilcoxon signed-rank test was conducted to determine differences in physical activity levels over time between baseline and post-intervention. Independent samples *t*-test was conducted to determine the mean differences for baseline, during-intervention and change values between the intervention group and the control group. Paired-samples *t*-test was conducted to determine the differences in means between baseline and during/post-intervention. ANCOVA was used to determine the differences in post-intervention means between the intervention group and control group after adjusting for baseline values. Pearson’s correlation test was run to assess the correlation between changes in anthropometric measures, iron status markers, hsCRP, and serum hepcidin. All reported *p* values were made based on two-tailed tests. Differences were considered statistically significant at values of *p* < 0.05.

## 3. Results

**Figure 1:**
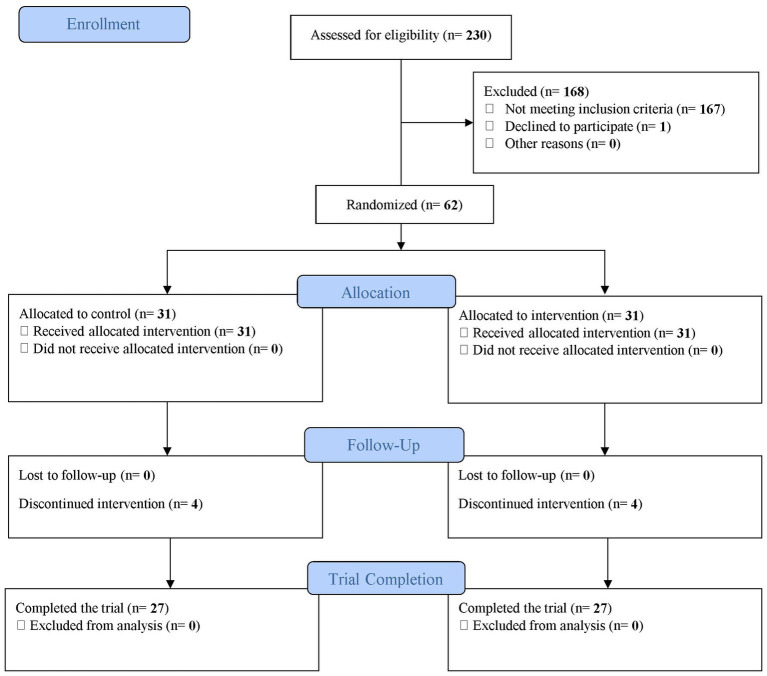
Flow chart illustrating the progress of the trial.

The study was conducted from March to September 2021. Overall, 230 women were screened, 167 did not meet the inclusion criteria, and one withdrew from participating. Sixty-two participants were randomized (1:1) into intervention and control groups and enrolled in the study. However, four participants from the intervention and four from the control group dropped out due to pregnancy. The final sample size who completed the study was 54 (27 from each group; Figure 1).

### 3.1. Baseline characteristics

The mean age of all participants was 26.5 ± 3.7. There were no significant differences in sociodemographic characteristics, anthropometric measurements (except waist circumference), and clinical data (except RBC count and hematocrit) between the intervention group and the control group at baseline (Table 1).

**Table 1:** Baseline characteristics of study participants.

Variables^*^	Intervention group (*n* = 31)	Control group (*n* = 31)	*p* value^**^
Age (years)	26.9 (3.9)	26.0 (3.6)	0.347
Education level			
Secondary school	12 (38.7%)	5 (16.1%)	0.094
Diploma degree	3 (9.7%)	7 (22.6%)	
Bachelor’s degree	16 (51.6%)	19 (61.3%)	
Marital status			
Single	7 (22.6%)	11 (35.5%)	0.263
Married	24 (77.4%)	20 (64.5%)	
Employment status			
Employed	5 (16.1%)	4 (12.9%)	0.665
Students	6 (19.4%)	9 (29.0%)	
Unemployed/housewives	20 (64.5%)	18 (58.1%)	
Family size	5.4 (1.9)	5.1 (2.2)	0.543
Household income			
Low (JD 250 or less)	4 (12.9%)	6 (19.4%)	0.146
Moderate (JD 251–500)	26 (83.9%)	20 (64.5%)	
High (JD 501–750)	1 (3.2%)	5 (16.1%)	
Height (cm)	160.6 (5.8)	159.7 (6.5)	0.53
Weight (kg)	86.7 (12.3)	83.7 (11.1)	0.316
BMI (kg/m^2^)	33.6 (4.8)	32.9 (4.5)	0.54
Body weight status			
Overweight	6 (19.4%)	7 (22.6%)	0.755
Obese	25 (80.6%)	24 (77.4%)	
Body fat (%)	42.4 (4.7)	39.9 (5.5)	0.06
WC (cm)	99.5 (11.8)	93.6 (9.0)	**0.031**
HC (cm)	118.8 (7.8)	115.1 (7.5)	0.063
WHR	0.84 (0.07)	0.81 (0.06)	0.183
RBC count (10^6^/μL)	4.7 (0.4)	5.0 (0.6)	**0.016**
Hemoglobin (g/dL)	11.2 (0.9)	11.3 (0.7)	0.415
Hematocrit (%)	36.1 (2.8)	37.7 (2.9)	**0.03**
MCV (fL)	75.1 (5.2)	74.7 (6.7)	0.794
MCH (pg)	24.1 (2.8)	23.3 (2.9)	0.29
MCHC (g/dL)	31.3 (1.4)	30.7 (1.4)	0.099
Serum ferritin (ng/mL)	9.3 (7.5)	9.7 (7.6)	0.837
Serum iron (μg/dL)	38.0 (19.8)	43.6 (18.9)	0.261
TIBC (μg/dL)	351.9 (58.0)	369.0 (53.3)	0.232
TS (%)	11.5 (6.8)	12.3 (6.4)	0.638
hsCRP (mg/L)	10.5 (9.9)	9.3 (10.4)	0.644
Serum hepcidin (ng/mL)	5.1 (3.2)	4.5 (3.4)	0.453

* BMI, body mass index; WC, waist circumference; HC, hip circumference; WHR: waist-hip ratio; RBC, red blood cell; MCV, mean corpuscular volume; MCH, mean corpuscular hemoglobin; MCHC, mean corpuscular hemoglobin concentration; TIBC, total iron binding capacity; TS, transferrin saturation; hsCRP, High-sensitivity C-reactive protein.

** Categorical variables were analyzed using Chi-squared test and expressed as numbers and percentages. Continuous variables were analyzed using independent samples *t*-test and expressed as means and standard deviations. *p* values < 0.05 are significantly different and presented in bold.

### 3.2. Changes in physical activity levels

About half of the participants in each group (intervention group and control group) had high physical activity levels at baseline and post-intervention. There were no significant differences in physical activity levels between the intervention group and the control group at baseline and post-intervention. Furthermore, there were no significant differences in physical activity levels over time between baseline and post-intervention for participants in the intervention group and the control group (Table 2).

**Table 2:** Physical activity levels of study participants at baseline and post-intervention.

Physical activity levels	Intervention group (*n* = 27)	Control group (*n* = 27)	*p* value^*^
Baseline (at day 90)			
Low	6 (22.2%)	7 (25.9%)	0.819
Moderate	8 (29.6%)	6 (22.2%)	
High	13 (48.1%)	14 (51.9%)	
Post-intervention			
Low	7 (25.9%)	9 (33.3%)	0.821
Moderate	5 (18.5%)	4 (14.8%)	
High	15 (55.6%)	14 (51.9%)	
*p* value^‡^	0.909	0.588	

* Chi-squared test was conducted to determine differences in physical activity levels between the intervention group and the control group. Values of *p* < 0.05 are significantly different.

‡ Wilcoxon signed-rank test was conducted to determine differences in physical activity levels over time between baseline and post-intervention. Values of *p* < 0.05 are significantly different.

### 3.3. Changes in dietary intake

Iron intake was significantly reduced in the intervention group (−2.2 ± 3.0 mg/day, *p* = 0.001). The mean of change in iron intake for the intervention group (−2.2 ± 3.0 mg/day) was significantly different from that reported for the control group (0.1 ± 1.9 mg/day; *p* = 0.001). Similar results were reported for the intake of energy, protein, carbohydrate, and fat (Table 3).

**Table 3:** Change in energy, macronutrients, fiber, and micronutrients intake of study participants.

Variables	Intervention group (*n* = 27)	Control group (*n* = 27)	*p* value	Recommended intake^#^ (Females, 19–30 years)
Energy (kcal/day)				−
Baseline (at day 90)	1,742 (355)	1,706 (394)	0.725^‡^	
During-intervention	1,323 (240)	1,708 (418)	**<0.001** ^‡^	
*p* value	**<0.001** ^§^	0.912^§^		
Change	−419 (203)	2 (108)	**<0.001** ^‡^	
Protein (g/day)				46 g/day
Baseline (at day 90)	61.7 (12.5)	63.1 (13.4)	0.696^‡^	
During-intervention	51.6 (16.1)	63.4 (16.8)	**0.011** ^‡^	
*p* value	**<0.001** ^§^	0.824^§^		
Change	−10.1 (12.1)	0.3 (6.4)	**<0.001** ^‡^	
Carbohydrate (g/day)				130 g/day
Baseline (at day 90)	225.9 (54.7)	208.3 (53.8)	0.238^‡^	
During-intervention	186.0 (49.8)	208.7 (57.4)	0.126^‡^	
*p* value	**<0.001** ^§^	0.907^§^		
Change	−39.9 (41.6)	0.4 (19.1)	**<0.001** ^‡^	
Fat (g/day)				Not determined
Baseline (at day 90)	67.4 (14.5)	70.3 (20.8)	0.548^‡^	
During-intervention	43.2 (15.0)	70.4 (22.7)	**<0.001** ^‡^	
*p* value	**<0.001** ^§^	0.975^§^		
Change	−24.1 (17.6)	0.1 (7.8)	**<0.001** ^‡^	
Fiber (g/day)				25 g/day
Baseline (at day 90)	16.9 (4.8)	14.6 (3.6)	0.062^‡^	
During-intervention	15.7 (9.2)	14.9 (4.4)	0.691^‡^	
*p* value	0.377^§^	0.631^§^		
Change	−1.2 (7.0)	0.2 (2.4)	0.322^‡^	
Iron (mg/day)				18 mg/day
Baseline (at day 90)	11.5 (3.9)	11.2 (3.6)	0.754^‡^	
During-intervention	9.3 (4.8)	11.3 (4.8)	0.129^‡^	
*p* value	**0.001** ^§^	0.751^§^		
Change	−2.2 (3.0)	0.1 (1.9)	**0.001** ^‡^	
Vitamin C (mg/day)				75 mg/day
Baseline (at day 90)	63.5 (38.4)	52.7 (23.0)	0.216^‡^	
During-intervention	64.6 (52.2)	50.4 (27.6)	0.218^‡^	
*p* value	0.874^§^	0.411^§^		
Change	1.0 (33.5)	−2.4 (14.8)	0.631^‡^	
Vitamin A (μg RAE/day)				700 μg RAE/day
Baseline (at day 90)	360.0 (348.8)	432.5 (497.3)	0.538^‡^	
During-intervention	306.9 (451.2)	366.3 (281.1)	0.564^‡^	
*p* value	0.358^§^	0.442^§^		
Change	−53.2 (295.0)	−66.2 (441.1)	0.899^‡^	
Vitamin D (μg/day)				15 μg/day
Baseline (at day 90)	0.27 (0.22)	0.27 (0.21)	0.960^‡^	
During-intervention	0.22 (0.21)	0.26 (0.22)	0.523^‡^	
*p* value	0.286^§^	0.690^§^		
Change	−0.04 (0.21)	−0.01 (0.12)	0.462^‡^	
Vitamin E (mg/day)				15 mg/day
Baseline (at day 90)	3.3 (2.6)	4.1 (2.7)	0.287^‡^	
During-intervention	2.1 (1.8)	3.9 (2.5)	**0.006** ^‡^	
*p* value	0.050^§^	0.456^§^		
Change	−1.2 (3.0)	−0.2 (1.7)	0.160^‡^	
Vitamin K (μg/day)				90 μg/day
Baseline (at day 90)	72.0 (125.9)	97.2 (119.4)	0.453^‡^	
During-intervention	94.9 (349.3)	88.6 (149.3)	0.932^‡^	
*p* value	0.660^§^	0.670^§^		
Change	22.9 (267.1)	−8.6 (103.7)	0.571^‡^	
Vitamin B1 (Thiamine; mg/day)				1.1 mg/day
Baseline (at day 90)	1.05 (0.46)	1.00 (0.38)	0.696^‡^	
During-intervention	0.87 (0.47)	1.01 (0.43)	0.241^‡^	
*p* value	**0.004** ^§^	0.699^§^		
Change	−0.18 (0.29)	0.01 (0.14)	**0.004** ^‡^	
Vitamin B2 (Riboflavin; mg/day)				1.1 mg/day
Baseline (at day 90)	0.79 (0.35)	0.86 (0.40)	0.465^‡^	
During-intervention	0.61 (0.40)	0.81 (0.27)	**0.033** ^‡^	
*p* value	**0.004** ^§^	0.317^§^		
Change	−0.18 (0.29)	−0.05 (0.25)	0.082^‡^	
Vitamin B3 (Niacin; mg/day)				14 mg/day
Baseline (at day 90)	13.0 (5.1)	13.2 (4.2)	0.918^‡^	
During-intervention	10.3 (5.7)	12.9 (4.4)	0.066^‡^	
*p* value	**0.003** ^§^	0.484^§^		
Change	−2.7 (4.3)	−0.3 (1.9)	**0.009** ^‡^	
Vitamin B6 (Pyridoxine; mg/day)				1.3 mg/day
Baseline (at day 90)	0.64 (0.27)	0.61 (0.21)	0.673^‡^	
During-intervention	0.59 (0.40)	0.58 (0.24)	0.886^‡^	
*p* value	0.478^§^	0.268^§^		
Change	−0.04 (0.30)	−0.03 (0.12)	0.807^‡^	
Vitamin B12 (Cobalamin; μg/day)				2.4 μg/day
Baseline (at day 90)	1.14 (1.02)	2.05 (4.79)	0.338^‡^	
During-intervention	0.78 (0.89)	1.15 (0.89)	0.140^‡^	
*p* value	0.135^§^	0.321^§^		
Change	−0.36 (1.20)	−0.90 (4.63)	0.555^‡^	
Biotin (μg/day)				30 μg/day
Baseline (at day 90)	5.08 (3.27)	4.61 (3.25)	0.596^‡^	
During-intervention	5.03 (4.57)	4.45 (3.24)	0.593^‡^	
*p* value	0.939^§^	0.762^§^		
Change	−0.05 (3.26)	−0.15 (2.63)	0.896^‡^	
Folate (μg/day)				400 μg/day
Baseline (at day 90)	267.3 (156.1)	238.6 (98.2)	0.422^‡^	
During-intervention	226.8 (201.4)	235.7 (101.8)	0.839^‡^	
*p* value	0.077^§^	0.725^§^		
Change	−40.5 (114.5)	−2.9 (42.3)	0.119^‡^	
Pantothenic acid (mg/day)				5 mg/day
Baseline (at day 90)	2.71 (1.42)	2.59 (1.06)	0.742^‡^	
During-intervention	2.21 (1.06)	2.54 (1.02)	0.253^‡^	
*p* value	**0.008** ^§^	0.520^§^		
Change	−0.50 (0.90)	−0.06 (0.46)	**0.029** ^‡^	
Calcium (mg/day)				1,000 mg/day
Baseline (at day 90)	542.1 (178.4)	545.2 (187.2)	0.951^‡^	
During-intervention	450.8 (188.3)	540.9 (241.3)	0.132^‡^	
*p* value	**0.027** ^§^	0.835^§^		
Change	−91.3 (202.9)	−4.3 (105.7)	0.054^‡^	
Magnesium (mg/day)				310 mg/day
Baseline (at day 90)	142.6 (52.9)	140.7 (44.1)	0.887^‡^	
During-intervention	125.9 (88.6)	142.1 (56.7)	0.426^‡^	
*p* value	0.206^§^	0.786^§^		
Change	−16.7 (66.8)	1.4 (27.3)	0.201^‡^	
Manganese (mg/day)				1.8 mg/day
Baseline (at day 90)	2.05 (0.87)	1.82 (0.77)	0.313^‡^	
During-intervention	1.85 (1.34)	1.90 (1.06)	0.880^‡^	
*p* value	0.246^§^	0.344^§^		
Change	−0.19 (0.85)	0.08 (0.45)	0.141^‡^	
Phosphorus (mg/day)				700 mg/day
Baseline (at day 90)	533.6 (221.6)	530.0 (189.3)	0.948^‡^	
During-intervention	427.5 (219.8)	525.0 (214.4)	0.105^‡^	
*p* value	**0.002** ^§^	0.767^§^		
Change	−106.2 (156.2)	−5.0 (86.7)	**0.005** ^‡^	
Potassium (mg/day)				2,600 mg/day
Baseline (at day 90)	1185.1 (385.6)	1133.5 (314.2)	0.592^‡^	
During-intervention	1096.5 (660.8)	1117.4 (339.8)	0.885^‡^	
*p* value	0.324^§^	0.574^§^		
Change	−88.5 (457.8)	−16.1 (147.3)	0.440^‡^	
Sodium (mg/day)				1,500 mg/day
Baseline (at day 90)	2648.0 (781.7)	2681.9 (660.6)	0.864^‡^	
During-intervention	2105.4 (543.1)	2712.9 (870.9)	**0.004** ^‡^	
*p* value	**<0.001** ^§^	0.765^§^		
Change	−542.7 (624.0)	31.0 (533.1)	**0.001** ^‡^	
Zinc (mg/day)				8 mg/day
Baseline (at day 90)	4.62 (1.67)	4.80 (1.88)	0.712^‡^	
During-intervention	3.65 (2.37)	4.73 (1.88)	0.069^‡^	
*p* value	**0.010** ^§^	0.716^§^		
Change	−0.97 (1.81)	−0.07 (0.99)	**0.029** ^‡^	
Fluoride (mg/day)				3 mg/day
Baseline (at day 90)	0.16 (0.07)	0.17 (0.13)	0.704^‡^	
During-intervention	0.12 (0.09)	0.17 (0.13)	0.091^‡^	
*p* value	0.052^§^	0.248^§^		
Change	−0.03 (0.08)	0.01 (0.03)	**0.026** ^‡^	
Copper (μg/day)				900 μg/day
Baseline (at day 90)	737.8 (325.8)	819.6 (583.9)	0.528^‡^	
During-intervention	578.5 (344.6)	703.3 (308.7)	0.167^‡^	
*p* value	**0.004** ^§^	0.272^§^		
Change	−159.3 (261.8)	−116.3 (538.8)	0.711^‡^	
Chromium (μg/day)				25 μg/day
Baseline (at day 90)	1.17 (0.63)	0.89 (0.68)	0.124^‡^	
During-intervention	1.03 (1.00)	0.96 (0.82)	0.772^‡^	
*p* value	0.531^§^	0.421^§^		
Change	−0.14 (1.13)	0.07 (0.43)	0.384^‡^	
Iodine (μg/day)				150 μg/day
Baseline (at day 90)	50.2 (27.5)	56.1 (45.6)	0.570^‡^	
During-intervention	40.2 (47.2)	57.2 (54.3)	0.225^‡^	
*p* value	0.260^§^	0.791^§^		
Change	−10.0 (44.9)	1.2 (23.1)	0.259^‡^	
Molybdenum (μg/day)				45 μg/day
Baseline (at day 90)	14.0 (21.6)	4.0 (7.6)	**0.030** ^‡^	
During-intervention	15.5 (45.2)	4.2 (10.2)	0.215^‡^	
*p* value	0.806^§^	0.716^§^		
Change	1.5 (32.0)	0.2 (3.0)	0.833^‡^	
Selenium (μg/day)				55 μg/day
Baseline (at day 90)	60.2 (31.6)	58.8 (25.5)	0.851^‡^	
During-intervention	49.5 (36.0)	58.7 (30.5)	0.314^‡^	
*p* value	**0.025** ^§^	0.987^§^		
Change	−10.7 (23.5)	−0.04 (12.3)	**0.043** ^‡^	

# Food and Nutrition Board, Institute of Medicine, National Academies: Dietary Reference Intakes (DRIs): Recommended Dietary Allowances and Adequate Intakes: https://ods.od.nih.gov/HealthInformation/nutrientrecommendations.aspx.

‡ Independent samples *t*-test was conducted to determine differences in baseline, during-intervention and change means between the intervention group and the control group. Values of *p* < 0.05 are significantly different and presented in bold.

§ Paired-samples *t*-test was conducted to determine differences in means between baseline and during-intervention for participants in the intervention group and the control group. Values of *p* < 0.05 are significantly different and presented in bold.

### 3.4. Changes in body weight and anthropometric variables

Body weight was significantly reduced in both groups. However, the weight loss was higher in the intervention compared to the control group (−7.4 ± 2.7 kg vs. −0.9 ± 1.5 kg). After adjusting for baseline values, the post-intervention weight mean for the intervention group (79.2 ± 12.1 kg) was significantly lower than the control group (83.5 ± 11.4 kg; *p* < 0.001). Similar results were reported for other anthropometric variables (BMI, body fat percentage, waist circumference, hip circumference, and WHR), except that changes in hip circumference and WHR were not statistically significant for the control group (Table 4).

**Table 4:** Change in anthropometric and clinical variables of study participants.

Variables^*^	Intervention group (*n* = 27)	Control group (*n* = 27)	*p* value
Weight (kg)			
Baseline (at day 90)	86.6 (12.7)	84.4 (11.5)	0.520^‡^
Post-intervention	79.2 (12.1)	83.5 (11.4)	**<0.001** ^#^
*p* value	**<0.001** ^§^	**0.004** ^§^	
Change	−7.4 (2.7)	−0.9 (1.5)	**<0.001** ^‡^
BMI (kg/m^2^)			
Baseline (at day 90)	33.6 (4.9)	33.0 (4.8)	0.666^‡^
Post-intervention	30.7 (4.6)	32.6 (4.7)	**<0.001** ^#^
*p* value	**<0.001** ^§^	**0.004** ^§^	
Change	−2.9 (1.1)	−0.3 (0.6)	**<0.001** ^‡^
Body fat (%)			
Baseline (at day 90)	42.6 (4.8)	39.7 (5.8)	0.050^‡^
Post-intervention	38.2 (5.3)	38.9 (5.9)	**<0.001** ^#^
*p* value	**<0.001** ^§^	**0.008** ^§^	
Change	−4.3 (2.0)	−0.8 (1.4)	**<0.001** ^‡^
WC (cm)			
Baseline (at day 90)	99.2 (12.1)	94.1 (9.4)	0.087^‡^
Post-intervention	86.6 (11.1)	91.2 (10.8)	**<0.001** ^#^
*p* value	**<0.001** ^§^	**0.015** ^§^	
Change	−12.7 (5.4)	−2.9 (5.7)	**<0.001** ^‡^
HC (cm)			
Baseline (at day 90)	119.0 (8.0)	115.6 (7.6)	0.124^‡^
Post-intervention	111.9 (9.6)	114.1 (10.0)	**<0.001** ^#^
*p* value	**<0.001** ^§^	0.075^§^	
Change	−7.1 (4.5)	−1.5 (4.1)	**<0.001** ^‡^
WHR			
Baseline (at day 90)	0.83 (0.07)	0.81 (0.06)	0.279^‡^
Post-intervention	0.77 (0.06)	0.80 (0.05)	**0.001** ^#^
*p* value	**<0.001** ^§^	0.088^§^	
Change	−0.06 (0.05)	−0.02 (0.04)	**0.001** ^‡^
RBC count (10^6^/μL)			
Baseline (at day 90)	4.7 (0.4)	5.1 (0.6)	**0.015** ^‡^
Post-intervention	5.1 (0.5)	5.0 (0.6)	**<0.001** ^#^
*p* value	**<0.001** ^§^	0.091^§^	
Change	0.4 (0.4)	−0.1 (0.3)	**<0.001** ^‡^
Hemoglobin (g/dL)			
Baseline (at day 90)	11.2 (0.9)	11.3 (0.8)	0.545^‡^
Post-intervention	11.7 (1.1)	11.4 (1.0)	**0.022** ^#^
*p* value	**<0.001** ^§^	0.246^§^	
Change	0.5 (0.6)	0.1 (0.6)	**0.022** ^‡^
Hematocrit (%)			
Baseline (at day 90)	36.0 (2.6)	37.6 (3.0)	**0.037** ^‡^
Post-intervention	37.2 (3.0)	37.0 (3.5)	**0.007** ^#^
*p* value	**0.006** ^§^	0.133^§^	
Change	1.2 (2.1)	−0.6 (2.0)	**0.002** ^‡^
MCV (fL)			
Baseline (at day 90)	74.8 (5.4)	74.1 (6.9)	0.709^‡^
Post-intervention	77.7 (7.5)	74.4 (7.4)	**0.005** ^#^
*p* value	**<0.001** ^§^	0.689^§^	
Change	2.9 (3.7)	0.2 (2.9)	**0.004** ^‡^
MCH (pg)			
Baseline (at day 90)	24.1 (2.9)	23.1 (3.0)	0.253^‡^
Post-intervention	25.3 (3.1)	23.4 (2.4)	**0.007** ^#^
*p* value	**0.001** ^§^	0.425^§^	
Change	1.2 (1.8)	0.2 (1.5)	**0.033** ^‡^
MCHC (g/dL)			
Baseline (at day 90)	31.3 (1.4)	30.7 (1.4)	0.115^‡^
Post-intervention	32.2 (1.2)	31.1 (1.2)	**0.021** ^#^
*p* value	**0.003** ^§^	0.277^§^	
Change	0.9 (1.5)	0.4 (1.6)	0.189^‡^
Serum ferritin (ng/mL)			
Baseline (at day 90)	9.9 (7.9)	9.6 (7.4)	0.913^‡^
Post-intervention	15.5 (10.4)	10.3 (7.7)	**<0.001** ^#^
*p* value	**<0.001** ^§^	0.321^§^	
Change	5.6 (5.8)	0.6 (3.3)	**<0.001** ^‡^
Serum iron (μg/dL)			
Baseline (at day 90)	37.2 (18.6)	44.2 (20.1)	0.191^‡^
Post-intervention	50.2 (22.4)	46.5 (24.2)	**0.025** ^#^
*p* value	**<0.001** ^§^	0.437^§^	
Change	13.0 (16.2)	2.3 (15.1)	**0.015** ^‡^
TIBC (μg/dL)			
Baseline (at day 90)	348.1 (57.4)	363.3 (52.1)	0.313^‡^
Post-intervention	330.7 (49.4)	360.7 (45.1)	**0.002** ^#^
*p* value	**0.003** ^§^	0.496^§^	
Change	−17.4 (27.4)	−2.6 (19.5)	**0.026** ^‡^
TS (%)			
Baseline (at day 90)	11.4 (6.6)	12.6 (6.7)	0.488^‡^
Post-intervention	16.0 (8.8)	13.3 (7.8)	**0.005** ^#^
*p* value	**<0.001** ^§^	0.447^§^	
Change	4.7 (5.6)	0.6 (4.4)	**0.005** ^‡^
hsCRP (mg/L)			
Baseline (at day 90)	10.1 (9.7)	8.8 (10.7)	0.650^‡^
Post-intervention	4.9 (6.0)	8.4 (7.7)	**<0.001** ^#^
*p* value	**<0.001** ^§^	0.706^§^	
Change	−5.2 (5.6)	−0.4 (5.9)	**0.004** ^‡^
Serum hepcidin (ng/mL)			
Baseline (at day 90)	5.8 (2.9)	5.1 (3.2)	0.435^‡^
Post-intervention	3.8 (3.0)	4.9 (2.8)	**0.006** ^#^
*p* value	**<0.001** ^§^	0.685^§^	
Change	−1.9 (2.2)	−0.2 (2.2)	**0.005** ^‡^

* BMI, body mass index; WC, waist circumference; HC, hip circumference; WHR, waist-hip ratio; RBC, red blood cell; MCV, mean corpuscular volume; MCH, mean corpuscular hemoglobin; MCHC, mean corpuscular hemoglobin concentration; TIBC, total iron binding capacity; TS, transferrin saturation; hsCRP, high-sensitivity C-reactive protein.

# ANCOVA test was conducted to determine differences in post-intervention means between the intervention group and the control group after adjusting for baseline values as a covariate. Values of *p* < 0.05 are significantly different and presented in bold.

‡ Independent samples *t*-test was conducted to determine differences in baseline and change means between the intervention group and the control group. Values of *p* < 0.05 are significantly different and presented in bold.

§ Paired-samples *t*-test was conducted to determine differences in means between baseline and post-intervention for participants in the intervention group and the control group. Values of *p* < 0.05 are significantly different and presented in bold.

### 3.5. Changes in clinical variables

The intervention group experienced a significant increase in hemoglobin (0.5 ± 0.6 g/dL), serum ferritin (5.6 ± 5.8 ng/mL), and serum iron (13.0 ± 16.2 µg/dL), and a significant decrease in hsCRP (−5.2 ± 5.6 mg/L), and serum hepcidin level (−1.9 ± 2.2 ng/mL) at the end of the trial. Conversely, changes in these variables were not statistically significant in the control group. After adjusting for baseline values, post-intervention means for hemoglobin (11.7 ± 1.1 g/dL), serum ferritin (15.5 ± 10.4 ng/mL), and serum iron (50.2 ± 22.4 μg/dL) for the intervention group were significantly higher than the control group (11.4 ± 1.0 g/dL, 10.3 ± 7.7 ng/mL, and 46.5 ± 24.2 μg/dL, respectively; *p* < 0.05). However, post-intervention means for hsCRP (4.9 ± 6.0 mg/L), and serum hepcidin level (3.8 ± 3.0 ng/mL) for the intervention group were significantly lower than the control group (8.4 ± 7.7 mg/L and 4.9 ± 2.8 ng/mL, respectively) after adjusting for baseline values (*p* < 0.05; Table 4).

### 3.6. Correlations between changes in weight and clinical variables

A significant negative correlation was observed between changes in weight and changes in RBC count (*r* = −0.529), hematocrit (*r* = −0.415), MCV (*r* = −0.293), MCH (*r* = −0.294), serum ferritin (*r* = −0.305), serum iron (*r* = −0.278), and TS (*r* = −0.308). Conversely, a significant positive correlation was observed between changes in weight and changes in TIBC (*r* = 0.290), hsCRP (*r* = 0.359), and serum hepcidin (*r* = 0.393; Table 5).

**Table 5:** Pearson’s correlation between changes in anthropometric measures, iron status markers, hsCRP, and serum hepcidin for all study participants (*n* = 54).

Variables^*^	RBC count (10^6^/μL)	Hemoglobin (g/dL)	Hematocrit (%)	MCV (fL)	MCH (pg)	MCHC (g/dL)	Serum ferritin (ng/mL)	Serum iron (μg/dL)	TIBC (μg/dL)	TS (%)	hsCRP (mg/L)	Serum hepcidin (ng/mL)
Weight (kg)	**−0.529**	−0.252	**−0.415**	**−0.293**	**−0.294**	−0.089	**−0.305**	**−0.278**	**0.290**	**−0.308**	**0.359**	**0.393**
BMI (kg/m^2^)	**−0.516**	−0.265	**−0.419**	**−0.293**	**−0.306**	−0.110	**−0.298**	**−0.285**	**0.270**	**−0.310**	**0.379**	**0.402**
Body fat (%)	**−0.534**	**−0.271**	**−0.457**	**−0.279**	**−0.350**	−0.220	**−0.281**	−0.230	**0.314**	−0.251	0.228	**0.466**
WC (cm)	**−0.385**	0.023	**−0.272**	−0.223	−0.105	0.026	−0.173	−0.056	0.146	−0.111	0.244	**0.312**
HC (cm)	**−0.347**	−0.186	**−0.272**	−0.230	−0.072	−0.087	−0.133	−0.148	0.103	−0.229	0.039	**0.361**
WHR	−0.223	0.148	−0.153	−0.134	−0.066	0.078	−0.126	0.022	0.149	0.003	0.259	0.154
hsCRP (mg/L)	0.007	−0.204	−0.116	−0.033	−0.132	−0.123	−0.197	−0.229	0.051	−0.204	−	0.068
Serum hepcidin (ng/mL)	−0.191	−0.198	−0.210	**−0.303**	−0.176	−0.260	−0.045	−0.234	0.236	−0.210	0.068	−

* RBC, red blood cell; MCV, mean corpuscular volume; MCH, mean corpuscular hemoglobin; MCHC, mean corpuscular hemoglobin concentration; TIBC, total iron binding capacity; TS, transferrin saturation; hsCRP, high-sensitivity C-reactive protein. Correlation coefficients are determined using Pearson’s correlation test. Correlation is statistically significant at value of *p* < 0.05 and significant correlations are presented in bold.

## 4. Discussion

In this study, we found that diet-induced weight loss among young women with overweight or obesity was associated with an improvement in iron indicators including a decrease in chronic inflammation and serum hepcidin level. There are few studies that previously investigated the effect of weight loss on iron status (37–40). One study was conducted among 15 obese children from Italy who participated in a 6-month weight loss program. By the end of that study, the children had a significant weight loss, a significant increase in iron absorption, and a significant decrease in serum hepcidin (37). In another study, children with overweight and obesity who participated in a school-based physical exercise study for 8 months showed a significant decrease in BMI z-score, C-reactive protein (CRP), and serum hepcidin, and a significant increase in serum iron (38). A study among young women with overweight and obesity showed that participants who achieved at least 10% weight loss had significantly higher mean serum iron and mean TS compared to those who lost less than 5% of baseline weight (39). Additionally, a recent study concluded that weight loss improved serum iron markers via a positive effect on low-grade chronic inflammation based on significant changes in body weight, CRP level, and iron markers among premenopausal Turkish women with overweight and obesity who participated in a weight loss trial (40). The findings of all the above-mentioned studies were in harmony with the current study results, which reported significant improvements in iron markers and a significant decrease in hsCRP and serum hepcidin.

Interestingly, our results revealed that diet-induced weight loss in the intervention group improved iron status despite a lower mean intake of dietary iron. A study reported that the iron supplement was less effective in improving iron status in children with high BMI-for-age *z*-scores (41). These findings indicate that iron status in individuals with overweight and obesity may be affected by chronic inflammation and hepcidin levels above and beyond dietary intake of iron although this requires further investigation.

Although a significant positive correlation was observed between changes in weight and changes in both hsCRP and serum hepcidin. The levels of hsCRP and serum hepcidin were not correlated. Current evidence proposed that the elevation in serum hepcidin associated with obesity is affecting iron absorption through inflammatory pathways (7). The regulation of hepcidin by inflammation occurs in response to certain pro-inflammatory cytokines such as interleukin-6. Interleukin-6 triggers hepcidin synthesis via signal transducer and activator of transcription 3-dependent pathways (42). In this study, only hsCRP is used as a measure of pro-inflammatory activity. Interleukin-6 could have a higher correlation with serum hepcidin.

To our knowledge, this is the first study conducted among participants with overweight/obesity and IDA using a RCT design. RCT is a rigorous study design used to examine cause-effect relationships between an intervention and outcome. In addition, we used the gold standard 7-day food record to measure dietary intake, which helped to reduce measurement bias. Nonetheless, this study was conducted at a single site, which may limit the population source; however, appropriate randomization techniques were applied to avoid bias. While metabolic measures such as serum glucose level and lipid profile were not included in this study, the use of only hsCRP to indicate chronic inflammation limited the outcomes. The intervention duration also involved festive seasons, which may affect the intervention and outcomes. Long-term studies of more than 3 months, using multi-centers, with participants from multi-ethnic backgrounds, and including interleukin-6 level as another indicator of pro-inflammatory activity are highly recommended for future studies. Nevertheless, the results have proven the importance of diet-induced weight loss to correct iron deficiency in individuals with overweight or obesity. This evidence could be used as the basis for the development of low-cost early treatment for IDA, as opposed to supplementation, which eventually will help reduce the overall treatment cost for health sectors.

## 5. Conclusion

Our findings indicate that diet-induced weight loss among young women with overweight/obesity and IDA was associated with an improvement in iron status and its related clinical markers. This effect was suggested to link with reduced chronic inflammation and serum hepcidin levels due to reduced intestinal iron absorption mechanism. This finding proves the positive effects of diet-induced weight loss and can be used as one of the bases for treatment guidelines in women at risk of IDA, particularly those overweight or obese.

## Data availability statement

The raw data supporting the conclusions of this article will be made available by the authors, without undue reservation.

## Ethics statement

The studies involving human participants were reviewed and approved by the Human Research Ethics Committee at Universiti Sultan Zainal Abidin, Terengganu, Malaysia (UniSZA/UHREC/2020/172) and Ajloun Health Directorate, Ministry of Health, Ajloun, Jordan (No.: 22/8/140). The patients/participants provided their written informed consent to participate in this study.

## Author contributions

NA, AA, and HA-J: conceptualization, methodology, validation, investigation, resources, supervision, project administration, and visualization. NA: software, formal analysis, data curation, writing—original draft preparation, and funding acquisition. AA and HA-J: writing—review and editing. All authors contributed to the article and approved the submitted version.

## Funding

This study was self-funded by NA (PhD Candidate).

## Conflict of interest

The authors declare that the research was conducted in the absence of any commercial or financial relationships that could be construed as a potential conflict of interest.

## Publisher’s note

All claims expressed in this article are solely those of the authors and do not necessarily represent those of their affiliated organizations, or those of the publisher, the editors and the reviewers. Any product that may be evaluated in this article, or claim that may be made by its manufacturer, is not guaranteed or endorsed by the publisher.

## Abbreviations

BMI: Body mass index
IDA: Iron deficiency anemia
GPAQ: Global physical activity questionnaire
WC: Waist circumference
HC: Hip circumference
WHR: Waist-hip ratio
RBC: Red blood cell
MCV: Mean corpuscular volume
MCH: Mean corpuscular hemoglobin
MCHC: Mean corpuscular hemoglobin concentration
TIBC: Total iron binding capacity
TS: Transferrin saturation
hsCRP: High-sensitivity C-reactive protein
CRP: C-reactive protein
RCT: randomized controlled trial
